# Public Health Impacts of Underemployment and Unemployment in the United States: Exploring Perceptions, Gaps and Opportunities

**DOI:** 10.3390/ijerph181910021

**Published:** 2021-09-23

**Authors:** Preethi Pratap, Alison Dickson, Marsha Love, Joe Zanoni, Caitlin Donato, Michael A. Flynn, Paul A. Schulte

**Affiliations:** 1 School of Public Health, University of Illinois Chicago, Chicago, IL 60612, USA; lovem@uic.edu (M.L.); jzanoni@uic.edu (J.Z.); cdonato@uic.edu (C.D.); 2 Champaign School of Labor and Employment Relations, University of Illinois Urbana, Chicago, IL 60607, USA; aquesada@illinois.edu; 3 National Institute for Occupational Safety and Health, Cincinnati, OH 45226, USA; dse4@cdc.gov (M.A.F.); pas4@cdc.gov (P.A.S.)

**Keywords:** underemployment, unemployment, health impacts, public health, decent work, United States

## Abstract

Background: Unemployment, underemployment, and the quality of work are national occupational health risk factors that drive critical national problems; however, to date, there have been no systematic efforts to document the public health impact of this situation. Methods: An environmental scan was conducted to explore the root causes and health impacts of underemployment and unemployment and highlight multilevel perspectives and factors in the landscape of underemployment and unemployment. Methods: included a review of gray literature and research literature, followed by key informant interviews with nine organizational representatives in employment research and policy, workforce development, and industry to assess perceived needs and gaps in practice. Results: Evidence highlights the complex nature of underemployment and unemployment, with multiple macro-level underlying drivers, including the changing nature of work, a dynamic labor market, inadequate enforcement of labor protection standards, declining unions, wage depression, and weak political will interacting with multiple social determinants of health. Empirical literature on unemployment and physical, mental, and psychological well-being, substance abuse, depression in young adults, and suicides is quite extensive; however, there are limited data on the impacts of underemployment on worker health and well-being. Additionally, organizations do not routinely consider health outcomes as they relate to their work in workforce or policy development. Discussion and Conclusions: Several gaps in data and research will need to be addressed in order to assess the full magnitude of the public health burden of underemployment and unemployment. Public health needs to champion a research and practice agenda in partnership with multisector stakeholders to illuminate the role of employment quality and status in closing the gap on health inequities, and to integrate workforce health and well-being into labor and economic development agendas across government agencies and industry.

## 1. Introduction

In the last thirty years the economic realities facing a majority of workers globally have become more severe due to broad shifts in macroeconomic policies and “disruptions involving new technologies and growing trade links” [1]. Often referred to as the “changing nature of work”, workers across the income spectrum increasingly face labor market insecurities and austerity in public programs designed to alleviate worker hardships [2,3,4,5,6]. These trends will likely continue as work becomes more precarious across numerous industrial sectors and worker protections, such as labor unions, are weakened [2]. Much has been written about the changing nature of work and growing labor market insecurities since the 1980s [2,3,4]. The changing nature of employment relationships and the contemporary “fissured workplace” [6] has resulted in a decline in the number of secure, full-time jobs [3,4,5]. There is a growing awareness that these economic forces may result in adverse effects on mental health, health behaviors, and chronic disease; however, there is a limited understanding of the full impact of the changing nature of work on the health and well-being of the workforce [7,8,9].

A renewed call for contemporary research to understand the relationship between health implications and unemployment, underemployment, and the changing nature of work has arisen in the past decade [10,11]. Rosenthal et al. [7] argue that because of “changes and cuts to social welfare services, decreases in the number of secure, full-time jobs, and general changes in the workforce over the past several years, it is important to re-examine the associations of employment status with mental and physical health, as they may differ from past decades”. Rosenthal [7] also suggests that it is “vital for the health and well-being of all people to increase not simply employment, but specifically full-time employment”.

The United States has experienced an alarming increase in suicide rates, opioid/other drug and alcohol abuse, and poorer physical and mental health, which can be traced in part to unemployment, underemployment, and the quality of working lives [12,13,14,15]. Research indicates that the absence of employment in good jobs contribute to these “deaths of despair” [12]. The lack of skills and opportunities and an increase in hopelessness and despair have led to a drastic increase in mortality arising in middle-age, white Americans [12], increased depression in young adults [13,14], the increased likelihood of unemployment and health problems in African Americans [13], and increased opioid overdose mortality associated with automotive assembly plant closures, “highlighting the role of declining economic opportunity in the US opioid overdose crisis” [15].

Underemployment and unemployment (and employment status in general) are often framed as economic problems, but they are also complex and multifaceted public health issues interacting with multiple social determinants of health that play a crucial role in perpetuating health inequities. Even before the COVID-19 pandemic, unemployment, underemployment, and the quality of work were national occupational health risk factors that drove critical national problems. The pandemic has made it very evident that the economy and jobs are social constructs that impact the distribution of health outcomes across a population [16,17]. Distribution of benefits and risks associated with work, just like the distribution of health outcomes, are inequitable and impacted by social factors, such as race and gender [18]. According to data from the Bureau of Labor Statistics [19], as of April 2021 the unemployment rate was 6.1 percent, and the number of unemployed persons, at 9.8 million, was still 4.0 million higher than in February 2020. The number of permanent job loses, at 3.5 million, is 2.2 million higher than February 2020. The number of long-term unemployed (those jobless for 27 weeks or more), at 4.2 million, is up by 3.1 million since February 2020 accounting for 43.0 percent of the total unemployed. It is anticipated that both the economy and jobs will continue undergoing significant changes and some jobs lost during the pandemic may be lost for good because businesses have adopted new ways to operate [1,20]. COVID-19 has only accelerated existing trends in remote work, e-commerce, and automation [1] and it may take several years to fully-realize the societal impacts of the new post-pandemic economy.

One key concept in public health is preventability, and there are conceivable ways to reduce and/or prevent the impacts of underemployment and unemployment. Work can be understood as a primary vehicle for responding to the *Surgeon General’s Call to Action on Community Health and Prosperity* [21]. The simple distinction of comparing employment to unemployment obscures the complexity of the relationship between work and health. Ezzy [22] and Warr [23] show that unemployment is not always harmful, and reemployment does not necessarily remediate underlying issues. Friedland and Price [24] succinctly summarized the research on these experiences: “when people lose jobs that are especially stressful, they often do not experience declines in well-being” [25] and “the quality of work plays a critical role in determining whether work is a source of well-being or cause of ill-being” [26]. Failure to account for the quality of employment may lead to an underestimation of both the harmful effects of unemployment and the beneficial effects of high-quality employment [27]; “Being unsatisfactorily employed is as bad as being unemployed” [28,29,30].

According to the International Labour Organization’s (ILO) definition, decent work “involves opportunities for work that is productive and delivers a fair income, security in the workplace and social protection for families, better prospects for personal development and social integration, freedom for people to express their concerns, organise and participate in the decisions that affect their lives and equality of opportunity and treatment for all women and men” [31]. During the United Nations General Assembly in September 2015, decent work and the four pillars of the Decent Work Agenda—employment creation, social protection, rights at work, and social dialogue—became integral elements of the new 2030 Agenda for Sustainable Development [32]. Goal 8 of the 2030 agenda calls for the promotion of sustained, inclusive, and sustainable economic growth, full and productive employment, and decent work, and will be a key area of engagement for the ILO and its constituents [32]. Furthermore, key aspects of decent work are widely embedded in the targets of many of the other 16 goals of the UN’s new development vision. While the United States is a large donor [33] and supporter of the ILO’s Decent Work Agenda, there is limited evidence of efforts to fully adopt or implement this agenda within the US. Decent work provides economic security, self-esteem, and social connectedness, and, as COVID-19 has highlighted, work can also undermine these same factors and contribute to the inequitable distribution of health outcomes [16,17,18].

Although there is an awareness of the impact of underemployment and unemployment on the health and well-being of US workers, to date there have been no systematic efforts to document the public health impact of this situation. The goal of this study was to gather evidence to reframe underemployment and unemployment (including, but not limited to, as it relates to a lack of employability skills) as public health problems and to contribute to the development of a strategic public health action agenda to address the identified areas of strengths, gaps, and opportunities. Systems thinking is a discipline for seeing wholes and interrelationships rather than linear cause–effect relationships. It involves a process of continuous change rather than perspectives at one point in time [34,35]. Applying a systems lens can reveal the causal pathways for underemployment and unemployment that exist at multiple levels within and outside organizations and institutions, and the resources and opportunities needed to address them. The authors conducted an environmental scan [36] with qualitative methods to “unpack” underemployment and unemployment through a public health lens to assess the challenges, needs, strongest areas of alignment, and possible leverage points for future research and action.

This study was completed just before the onset of the pandemic and the findings describe the pre-pandemic state of underemployment and unemployment in the US workforce by exploring the perspectives and interrelationships of stakeholders. However, these findings also forecast the fractured experience and challenges faced by workers and stakeholders during the on-going pandemic, and highlight the urgency in accounting for the growing impacts of underemployment and unemployment on the health and well-being of the workforce as we navigate the post-pandemic economy and future of work.

### Objectives

The primary objectives of this study were to: (1) describe the root causes/drivers of underemployment and unemployment; (2) document perceptions of underemployment and unemployment among multilevel stakeholders alongside the perceived barriers and facilitators to employment; (3) describe health and economic impacts of underemployment and unemployment; and (4) identify connections and points of leverage for action. 

## 2. Materials and Methods

### 2.1. Literature Review

Multiple comprehensive and structured searches of the scholarly literature were conducted between October 2018 and December 2019 in the following databases, without date restrictions: PubMed, Business Source Premier, CINAHL, EconLit, PsycINFO, Sociological Abstracts, and Web of Science Core Collection. Several iterative searches were performed using search terms centering on the two conceptual domains of underemployment/unemployment and health outcomes (health care services, mental health, depression, anxiety, suicide, addiction, food insecurities, homelessness, interventions, program evaluation, program implementation, skills gap, public health, community health, economy, labor policies). No specific exclusion criteria were used for this phase of the search. Separate searches on a number of subtopics were carried out in order to address the multiple facets of this broad topic. Subtopic searches included, but were not necessarily limited to: how underemployment and unemployment are defined in the literature, drivers of underemployment and unemployment, health and economic implications at both the individual and community levels, facilitators and barriers to employment, and key stakeholders in addressing these issues. General web searches were performed to identify gray literature, as well as targeted searches of the websites of organizations known to be working on addressing issues of underemployment and unemployment. These searches included, but were not limited to, policy groups such as the Economic Policy Institute and the National Bureau of Economic Research, the Harvard Business Review, the International Labour Organization, the World Health Organization, J.P. Morgan Chase, the National Employment Law Project, the Bureau of Labor Statistics, the Organisation for Economic Co-operation and Development, the National Institute for Occupational Safety and Health, the National Skills Coalition, the US Department of Labor, and federal, state, and local workforce training groups. Literature retrieved included think tank and research reports, white papers, and US national, state, and local government reports. A total of 327 articles were amassed according to the methods outlined above (these included 286 peer-reviewed publications and 41 gray literature publications). The abstracts were scanned by three team members (including a university librarian) and sorted by the following themes: health impact, mental health, community and social aspects, skills gaps, economics, policies and programs related to skills training, public–private workforce partnerships, underemployment, and unemployment. Two coders initially scanned articles and reports that they were familiar with from the literature and identified additional articles which were cited. A memoing process [37] was used to identify recurring themes related to the a priori research questions, which led to a comprehensive review of 52 articles and a basic examination of an additional 45 articles (including 13 gray literature publications). The authors prioritized US-specific publications and reports for this phase of the review; however, a few key studies from other developed countries, such as the United Kingdom, were included to support literature findings or draw comparisons. Initial results of the literature review were used to identify domain areas for the interviews.

### 2.2. Interviews

Representatives from nine different organizations were individually interviewed between March and September 2019, with each interview lasting approximately 45–60 min. Key informants included representatives from two nonprofit policy think tanks, two workforce development organizations, a national skills coalition, a grassroots worker center focusing on temporary staffing workers, one community colleges system (amongst the largest in the United States) and two industry representatives, one from a regional hotel and lodging trade association and one from a national manufacturing association. Key informants were identified from a pool of local, regional, and state, and national nonfederal organizations who are addressing worker health and safety, specifically through initiatives addressing underemployment and unemployment in the United States. An initial purposive sample of potential participants and their contact information was generated by the project team. The participants were then recruited to ensure that we captured a wide range of responses that illustrate the diverse interests and possible commonalities in addressing underemployment and unemployment and its connection to health. The participants selected were chosen to maximize understanding by increasing the likelihood of gaining access to perspectives that challenge, alter, and further inform our initial understanding of these issues from the literature.

A predetermined, semi-structured interview tool was used to elicit respondent beliefs and concerns about underemployment and unemployment and the impact on health. The interview tool was structured to cover specific topics, including: (1) how the concepts of underemployment and unemployment are perceived and characterized (including drivers) by stakeholders at the local, regional, and national levels; (2) health and its relationship to underemployment and unemployment; (3) initiatives currently underway that identify and address possible pathways to employment and the barriers to implementation of these initiatives; and (4) strengths and opportunities for engaging multisector stakeholders to align and increase resources to address underemployment and unemployment. Respondents were asked to express their opinions and concerns on these topics.

Analysis of the transcripts was conducted using qualitative content analysis (thematic), a method used to categorize large amounts of text from transcripts by assigning initial codes to text segments based on attributes to the phenomenon that accurately reflected the a priori research questions and relevant research findings [38]. Two coders reviewed and coded each interview transcript based on the focused coding categories: the perspectives on the definitions of underemployment and unemployment; drivers of each; health factors connected to the ability to access, or not access, work and livable wages (an approximate income to meet a family’s basic needs [39]); how they view work as a meaningful and significant determinant of health outcomes; perceptions about skills gap in the labor market; barriers to doing the work they are doing; and emergent themes (i.e., those unanticipated issues relevant to the topic that participants raised). An intra-case/cross-case analysis was performed to analyze and code both the anticipated (a priori) and unanticipated themes. Relevant quotations thus organized were selected for further analysis (based on the initial coding and alignment with research objectives).

All phases of coding and analysis were performed manually. Data were analyzed through an iterative process of reflective reading of transcripts, interpretive memoing, and coding and exploring inconsistencies and divergent perspectives, or cases that did not fit with the initial interpretations [37]. Respondents’ accounts of their experiences working in policy, advocacy, workforce development, and other relevant labor market issues were then clustered together in themes to provide an account of respondent meanings and interpretations of the phenomenon [37]. The open-ended questions used to guide the interviews elicited a variety of participant responses, and qualitative analysis uncovered an assortment of main themes. Key themes from the literature review and key informant interviews were examined to identify a crosswalk of common and divergent themes, areas of alignment, and gaps in evidence [38]. The uniformity of themes across interviews seems to suggest that the results are reasonably reliable.

## 3. Results

The findings presented in this section highlight the four overarching themes and several subthemes emerging from the crosswalk of themes from the review of 74 peer-reviewed and gray literature publications and the nine key informant interviews. These themes describe the landscape of unemployment, underemployment, and the impact on health outcomes.

### 3.1. Large Macro-Level Issues Are Driving the Underemployment and Unemployment Narrative

**Figure 1:**
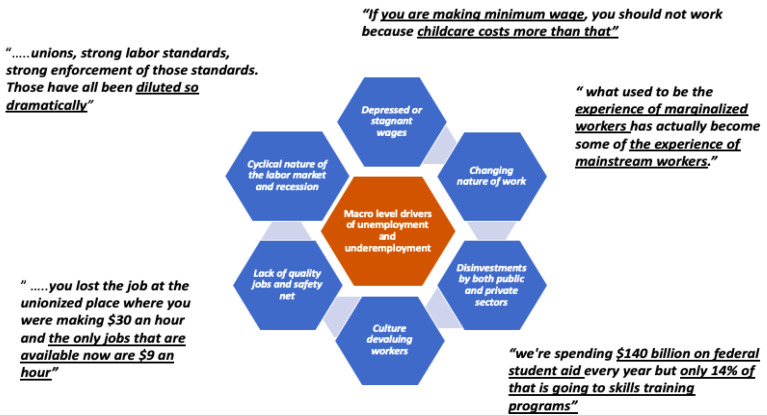
Description of the key themes emerging from the literature review and key informant interviews.

A number of convergent themes emerged from the literature review and discussion around the significant drivers of underemployment and unemployment. While the cyclical nature of the labor market and recession were commonly reported drivers of underemployment and unemployment, a number of other factors, (Figure 1) including the changing nature of work [40], a lack of quality jobs [41], the devaluing of workers, depressed wages, disinvestments by both the public and private sectors on worker education and training [6], a dynamic labor market, and inequalities in the distribution of public funding/resources emerged in both the literature and key informant interviews.

Fenwick and Tausig [42] argued that we need to address the role that macroeconomic changes have had on the structure of work. As economic changes transformed the organization of work, they modified the work environment, subjecting employees to stressful work conditions. They also linked the economic stress and work stress approaches by arguing that “macroeconomic and economic changes affect exposure to life events such as unemployment as well as the level of stress from changing work roles and the structure of the workplace” [42,43]. Ferman and Gordus [44] proposed a model in which economic change resulted in displacement and loss of work for some groups. In their study, they observed that “displaced individuals may accept employment with fewer economic and other benefits including protection from unfair work practices, or lower status positions…” and that “career patterns of these individuals may change, as they find immediate re-employment, move from job to job, or remain unemployed, and this was argued to cause stress and instability” [44]. 

Research also explores the wage penalty suffered by involuntary part-time workers. Golden [45] found that amongst workers paid by the hour in the US, voluntary part-time workers earned USD 15.61 per hour on average compared with only USD 14.53 for those who could “find only part time work”. Using data from the United Kingdom, Blanchflower and Bell obtained similar results in their working paper [46], finding that individuals who wanted more hours (part-timers wanting full-time employment), had lower hourly wages than voluntary part-timers and full-timers. This leads to the implication that “part-timers who want extra hours are paid less than part-timers who are content with their hours”. In the US, there was a norm of average wages rising 4 percent per year in the years before the Great Recession (2007–2009) and 2 percent subsequently. The US federal minimum wage has remained unchanged at USD 7.25 since 2009 [47]. Twenty-nine states have set the minimum wage above the federal minimum wage [47]. Research suggests associations of increased wages with improved birth outcomes, lower rates of sexually transmitted infections among women, as well as decreases in both suicide mortality and hypertension [48,49,50,51].

One often cited explanation for persisting underemployment and unemployment is the debate over the supposed “skills gap” asserted by US employers. The literature revealed diverse views of the skills gap argument in the academic literature [52,53,54,55,56] versus the consulting world [57,58,59]. Data from reports (mostly industry specific) in the gray literature and some of the key informant interviews support the fact that there are some skills gaps, but there was no strong evidence to support the argument that there is an overall skills gap; rather, what emerged was a skills mismatch issue, a lack of clear information on what the education and skills acquisition pathway is for the future of work, and employers’ lack of investment in skills training for their workers.

Five out of nine interview respondents described the current labor economy as set up to basically discount the value of workers and lamented the role of declining unions and enforcement standards and their impact on job quality and pay:

*“All of the things that gives power to workers……like unions, strong labor standards, strong enforcement of those standards. Those have all been diluted so dramatically…”*

—interview respondent from a policy group.

*“you lost the job at the unionized place where you were making USD 30 an hour and the only jobs that are available now are USD 9 an hour. It’s just like totally and utterly demoralizing and catastrophic at every level. […]…… where are those other good jobs you can find when you lost a job…[…]… one of the things that made it so bad for so many people is they can’t find another one that can sustain their living standards”*

—interview respondent from a workforce development organization.

*“what used to be the experience of marginalized workers (for whatever reason they were marginalized) has actually become some of the experience of mainstream workers”*

—interview respondent from a workforce development organization.

*“Employers have no investment in their workers. They’re not the employer of record anymore […]…they have every incentive in the world to outsource their work. So, it doesn’t matter if you’re blue collar, white collar or even university professors. […]……work is constantly being non-standardized”*

—interview respondent from a worker advocacy group.

*“the jobs aren’t particularly good. I just don’t know people who are like, “Oh, I’m dying to be in retail.”… the growth in terms of number of jobs…it is in the services, it is in hospitality, retail, education services, and the occupations that grow the most are the lowest paid. I think the wages thing is totally key. We have the employer, who in this country has a lot of power to keep wages down. And they do exercise that power”*

—interview respondent from a policy group.

Among the interview respondents, there was a strong convergence perception that the idea of a skills gap preventing access to the labor market, a claim made by many employers as the most significant barrier to work, is only one explanation for barriers to the labor market. Six of the nine respondents pointed to the role of particularly burdensome barriers (unrelated to skills) for certain individuals and communities in accessing the labor market, including older age, history of incarceration, child/family care needs, transportation needs, and driver’s license status, all of which can significantly impede access to employment.

*“I think that the story is a lot less about workers not having the right skills. I’m not saying that skills are not important but a huge share of jobs in this country require no training or maybe some high school ……[…]… look at BLS data…and the astounding number of jobs that just don’t actually require that many specific skills. And the difference between what is a good job and not a good job are unions and labor standards. …[…]… is a bigger player than the skills story…”*

—interview respondent from a policy group.

Interview respondents also expressed concerns over the disproportionate amounts of both public and private investment going to people higher along the education spectrum.

*“we are not targeting that federal or private investment in workers who need it in order to progress along their career pathway. …[…]… we are spending USD 140 billion on federal student aid every year but only 14% of that is going to skills training programs, there’s a mismatch between what people are experiencing, and what people want to be doing and what we’re actually spending money on”*

—interview respondent from a skills coalition group.

Overall, interview respondents reported that underemployment and unemployment are influenced by a variety of factors that are often ignored by many policy makers and employers, yet have a direct impact on the ability of many people to access the labor market and gain the stability and health outcomes connected to attaching to the labor market in a sustainable way.

### 3.2. Gaps Exist in the Definitions and Measures of Underemployment and Unemployment

A common theme emerging from both the literature review and key informant interviews was that while the official unemployment rate was at historically low levels in the US prior to the pandemic, the large number of underemployed workers indicates that the country’s labor markets were not anywhere close to full employment, even before the pandemic. The Bureau of Labor Statistics (BLS) [60] defines full employment as an economy in which the unemployment rate equals the nonaccelerating inflation rate of unemployment (NAIRU), no cyclical unemployment exists, and GDP is at its potential. The full-employment assumption links BLS projections to an economy running at full capacity and utilizing all of its resources [60]. In order to best understand the negative health impacts linked to a lack of work, researchers are best served looking at the different measures of underemployment and unemployment and its impacts on varying geographies, sectors, and worker demographics. 

The BLS has a specific definition of unemployment: those who do not have a job but are available for work and have looked for work in the past four weeks. According to BLS data, in December 2019 (before the pandemic), the US unemployment rate was 3.5 percent, the lowest since 1969, and the number of unemployed persons was at 5.8 million [61]. Although the number of long-term unemployed (those jobless for 27 weeks or more) edged down to 1.2 million (20.9 percent of the unemployed), this number still remained higher than it was before the Great Recession (2007–2009) [61]. Similarly, the proportion of people unemployed for longer periods (52 weeks or more) also remained higher than before the Great Recession and about 1.2 million persons were marginally attached to the labor force in December 2019 [61]. These individuals were not in the labor force, wanted and were available for work, and had looked for a job sometime in the prior 12 months. They were not counted as unemployed because they had not searched for work in the 4 weeks preceding the survey. Among the marginally attached, there were 277,000 discouraged workers in December 2019, which was 98,000 less than the number of such workers in the previous year [61]. Discouraged workers are persons not currently looking for work because they believe no jobs are available for them. The remaining 969,000 persons marginally attached to the labor force had not searched for work for reasons such as school attendance or family responsibilities [61]. 

As defined by Feldman [62], underemployment is the inability to obtain adequate employment relative to some standard. Based upon previous research conducted by Feldman [62] and McKee-Ryan and Harvey [63], Thompson et al. [64] aggregated the types of underemployment that affect how individuals perceive their job situation. Accordingly, education or experience underemployment occurs when individuals possess more education or experience than is required for their jobs, while “individuals who earn less at their current job than they did previously or those earning 20% less than their peers suffer from wage underemployment” [64]. Job status underemployment characterizes individuals who would like to work full time but who can only find part-time employment, and job field underemployment affects people who are forced to work in a field of employment that is outside their area of formal education and/or job experience [64].

The most widely available measure of underemployment estimated by statistical agencies around the world, such as the BLS in the US, the Office for National Statistics in the UK, and the EU statistical agency Eurostat, is the share of involuntary part-time workers in total employment, also known as the involuntary part-time rate (IPTR). In Europe, involuntary part-timers are described as part-timers who want full-time jobs (PTWFT). In the United States they are described as part-time for economic reasons (PTFER). The number of PTFER, sometimes referred to as involuntary part-time workers (or underemployed workers) was at 4.3 million in the US in December 2019 [61], representing 2.7 percent of the working population. These individuals, who would have preferred full-time employment, were working part time because their hours had been reduced or they were unable to find full-time jobs. The fact that millions of workers wanted to work more hours indicated that the labor market still had plenty of slack. Gallup polls from 2010 found that 18 percent to 25 percent of American workers perceived themselves as underemployed—a stark contrast to the BLS underutilization rate of 6.4 percent [65,66]. Previous research by Jensen and Slack [67] concurs that “underemployment was proposed as a more exhaustive and hence more valid measure of employment hardship than mere unemployment.” Therefore, in December 2019, while the US unemployment rate had returned to levels seen pre-recession, underemployment remained elevated [61] and undercounted because, although people talk about the term “underemployment”, there is not much in the literature or policy about standard ways to measure it in the labor market. The US Labor Department, in its current measure of “part time for economic reasons”, does not ask how many hours the underemployed want to work and therefore does not do a good job of measuring underemployment.

The literature on the characteristics of underemployed individuals has grown exponentially in the past three decades. Blanchflower and Levin [68] showed that in the US the rise of underemployment “represents another dimension of labor underutilization.” Discrimination in the labor market based on age and race emerged as prominent themes in the literature. Early studies examining underemployment and the experiences of different racial and ethnic groups found that minority groups in the US (e.g., Blacks, Latinos, immigrants) have higher prevalence (compared to non-Hispanic Whites and Asians) of underemployment, even after controlling for human capital, industry, occupation, family structure, and other predictors [69,70,71,72]. Valletta et al. [73] found that young workers under the age of twenty-four, the single, the least educated, Blacks and Latinos, and the unincorporated self-employed are most likely to work part time involuntarily. Another early study focused on gender disparities, with women showing higher prevalence of working poverty and involuntary part-time work [74]. The overrepresentation of women, ethnic/racial minorities, and foreign-born individuals among the unemployed and underemployed suggests that structural disadvantages overlap and interact with one another in the lives of many workers, leading to cumulative precariousness that likely aggravates existing health inequities [75,76].

Key informants were first asked to provide their own personal definitions of the terms (underemployment and unemployment). Respondents shared the meaning, significance, and outcomes of varied perceptions about the definitions of underemployment and unemployment. Comments from respondents reveal miscommunication of unemployment rates provided to the public, influencing decisions and considerations from the public and policy makers. Most described a common public perception that while unemployment numbers may look good overall, substantial differences are experienced in the labor market not related to the economy or recessions, but based on factors related to age, race, immigrant/refugee status, trauma history, parenting status, caregiving/family/child responsibilities, history of incarceration, food insecurity, poverty, transportation access, driver’s license status, literacy skills, status of protections afforded from participation in unions, enforcement of labor standards, disability status, presence of addictions (with the individual or within the family), and homelessness.

*“…there are many ways to measure unemployment and there’s the standard rate that gets reported in the mainstream media every month. A lot of people think the more accurate analysis of the strength of the economy is the employment to population ratio ……And we are actually lower by historical standard in the employment to population ratio. Another standard is for people who are still unemployed (long-term unemployment)…[…]…usually post-recession in a recovery the length of long-term unemployment starts to shorten and that hasn’t been happening”*

—interview respondent from a policy group.

*“unemployment is at 3.5 percent. …..the overall numbers have enormous variation…….and enormously different outcomes for different individuals and different groups. Like the demographics and certain classes of workers. And the racial/minority unemployment rate is almost vastly different … essentially the black unemployment rates is twice the white unemployment ratings this time or that time”*

—interview respondent from a workforce development organization.

*“unemployment rate…is a kind of a marker that people are familiar with. For those of us in the field, we know that […] marker that people talk about globally is actually not a full picture. So that number doesn’t necessarily encompass all the folks out there who aren’t working. …we just need to make sure that people understand that”*

—interview respondent from a policy group.

*“Underemployment for me is really just about folks that are working in subpar economic scenarios……underemployment could also then be defined as working multiple jobs to barely make it, or working multiple jobs to make it… but being underemployed in each of those areas makes it more difficult to participate say fully in family or whatever the circle of peoples experience is…”*

—interview respondent from a community colleges system.

### 3.3. Need for Quality Data on Health Outcomes for Individuals and Communities by Demographics and Geographic Levels; and for Connecting Health Data to Policy Makers, Workforce Development Agencies, Employers and Funders

#### 3.3.1. Extensive Empirical Literature on Impacts of Unemployment (Mostly Long Term) on Physical, Mental Health and Psychological Well-Being

The connection between unemployment and negative health outcomes has been well-established in the academic literature [77]. For the most part, unemployment, particularly episodes that last longer than six months, is associated with significant detrimental effects in psychological and physical well-being [78,79]. Perhaps most profound is how unemployment has been linked with increased mortality [80,81,82,83,84,85,86,87,88,89]. A number of studies have demonstrated the link between unemployment and mental health problems, including depression, somatization, anxiety, and substance abuse [90,91,92]. In their three-wave panel study on the relationships between auto plant closings and unemployment of blue-collar workers, Broman et al. explored how the mental health impacts of long-term unemployment vary across race and gender [93]. Specifically, they found that the “effect of long-term unemployment on distress and drinking was more severe among less educated workers, and responses of blacks were especially sensitive to level of education” [93]. A number of studies have also found a correlation between unemployment and increased smoking and alcohol usage [94,95,96].

A 2003 study conducted by Brown et al., from the CDC National Center for Chronic Disease Prevention and Health Promotion, examined the relationships between unemployment and frequent mental distress (FMD), defined as 14 or more mentally unhealthy days during the previous 30 days, in a study of over 98,000 men and women aged 25–64 years in the United States [97]. This team of researchers discerned similar patterns across gender, race/ethnicity, education, income, and high unemployment areas of the US, and concluded that “unemployed persons are a population in need of public health intervention to reduce the burden of mental distress”, and that “public health officials should work with government officials to incorporate the health consequences of unemployment into economic policymaking” [97]. In 1986, Dooley and Catalano found that communities with high rates of unemployment have higher rates of suicide and mental hospitalization [98]. Research published in 2011 by Luo et al. examined the relationships between overall and age-specific suicide rates and US business cycles from 1928 to 2007 [99]. Results from their analyses showed that the “overall suicide rate generally rose during recessions and fell during expansions”, while “age-specific suicide rates responded differently to recessions and expansions” [99]. In conclusion, researchers found that business cycles (and thus unemployment levels) have the propensity to affect suicide rates, although different age groups responded differently, and that “public health responses are a necessary component of suicide prevention during economic downturns” [99].

McGee and Thompson [13] used data from the 2010 Behavioral Risk Factor Surveillance System (BRFSS) to specifically analyze the relationship between unemployment and symptoms of depression among emerging adults (aged 18 to 25 years). Findings from their research show that almost 12 percent of emerging adults were depressed and about 23 percent were unemployed, and that significantly more unemployed than employed young adults were diagnosed with depression. In summary, their models concluded that the “odds of depression were about 3 times higher for unemployed than employed emerging adults” [13]. While many factors can contribute to depression, unemployment is typically associated with high rates of depression among adults [78,100] due in part to losses in social contact and status or stress related to income loss [101]. Earlier research by Galambos et al. [102] established that for emerging adults, “long experiences of unemployment increase the likelihood of experiencing depression throughout the transition” [102].

Moller [103] also noted that not all unemployed persons will seek medical assistance and that all policy initiatives must always take note that society’s most disadvantaged populations are likely to suffer most from unemployment, and that these groups might have more difficulty in accessing services and support programs [103,104]. A 2013 study [105] also drew the link between changes in the unemployment rate and health care choices among the affected. Specifically, Tefft et al. found that a 1 percent increase in state unemployment corresponded to a 1.58 percent reduction in the use of preventive health care services for women such as mammograms, pap tests, and annual check-ups [105]. Study researchers discerned that women and people who are already economically disadvantaged were especially sensitive to economic fluctuations. This is in part due to the fact that, as the authors noted, preventive medical care is underutilized in the United States, with only about half of the population following recommended guidelines. They suggested that “preventive care can carry a relatively high one-time cost and may be more likely to be seen as a relative luxury; therefore, changes in income, or fears of an impending loss of income during a downturn, may have a greater effect on their use” [105]. 

#### 3.3.2. Limited Research on Impacts of Underemployment on Physical, Mental and Psychological Well-Being

While the health effects of unemployment have been studied for a long time, the impacts of underemployment are less studied. The limited research in this area suggests that underemployment also negatively impacts health. Friedland and Price characterize “underemployment as a potential social stressor that places demands on workers and may compromise their health and well-being” [24]. Accordingly, “social stress is arousal due to an imbalance between perceived environmental demands and perceived capacity to respond to those demands” [24,106]. Using longitudinal data for the US from the Americans’ Challenging Lives study from 1986–1994, Friedland and Price [24] observed some support for the hypothesis that underemployment is related to lower levels of physical health. People who are status-underemployed report more chronic disease and less functional health than do adequately employed workers. People who are income-underemployed report less functional health than do adequately employed workers. Friedland and Price caution that the “relationship between underemployment and health and psychological well-being varies by both types of underemployment and indicator of health and wellbeing” [24]. They advise researchers to specify types of underemployment and specific health indicators when describing their relationships, rather than relying on generalizations.

In their July 2018 working paper, Blanchflower and Bell examined the intersection between underemployment and well-being using data from the UK [107]. The five main measures they used to measure well-being include: happiness; life satisfaction; whether life is worthwhile; anxiety and depression. Their research found that “those who wished to increase their hours (the underemployed) rose sharply in the years after 2008, while the number who wished to reduce their hours (the overemployed) fell slightly” and that post-recession, “over-employment fell back to pre-recession levels, but underemployment did not”. Blanchflower and Bell found that while the underemployed have higher levels of well-being than the unemployed and disabled, they have lower levels of well-being than any other group of workers, full- or part-time. Both unemployed and underemployed individuals were unhappy and depressed, due in large part to their loss of earnings and the coinciding lower standards of living. Blanchflower and Bell found that the fear of unemployment was on the rise, and also found evidence of a large rise in anxiety and depression among the underemployed (between 2008–2018), especially underemployed women [107]. Research by Jones-Johnson [108] suggests a link between underemployment and both psychological and interpersonal stress that may be particularly severe when minority group status is added to the mix.

Wooden, Warren, and Drago [109] and Wilkins [110] used longitudinal data from the 2001 Household, Income and Labour Dynamics in Australia (HILDA) survey, and found that underemployment lowers job and life satisfaction. Income and hours underemployment have also been shown to affect the health and well-being of workers. Prause and Dooley [111] found that underemployment lowered individuals’ self-esteem, while the following year, Dooley and Prause [112] found that workers who experience chronic hours or income underemployment report increases in symptoms of alcohol abuse. Workers who become hours- or income-underemployed after leaving high school report lower self-esteem than those who become adequately employed [111]. Finally, workers who moved from adequate employment into income underemployment experienced increases in depression, while underemployment by hours did not increase depression [27].

#### 3.3.3. Organizations do not Routinely Consider Health Outcomes as They Relate to Their Work in Workforce Development, Policy Development or in Programs Addressing Underemployment and Unemployment

All nine interview respondents described how their work does not primarily focus on health outcomes as it relates to underemployment and unemployment. When specifically asked how health relates to underemployment and unemployment, six out of nine respondents mentioned that they believe mental as well as physical health suffers in the face of unemployment or underemployment because of feelings of worthlessness, the stress of the instability, and/or the trauma associated with the exploitation experienced when employees work for abusive employers for the purpose of being able to take care of their families, but have no recourse and no support for their situation.

Respondents described being aware of unemployment related to mental health issues and post-traumatic stress disorder, and that mental health issues and addiction issues are often part of the reasons why adult workers have not been able to successfully attach to the labor market; however, organizations are limited in the knowledge and ability to measure or address these health and trauma-related impacts.

*“We don’t have any kind of connections to the health impact of a worker of not having a job. That’s not a lens that we look at”*

—interview respondent from industry.

*“Unfortunately, the link between unemployment and health and how unemployment might cause ill health is really not something that we’ve done any work on ourselves. It’s a little bit peripheral to what we have typically looked at but I agree that we should better understand what the health impacts are”*

—interview respondent from a policy group.

*“So much of your identity is tied up in what you do for a living and how you take care of your family that I think that mental health is a natural impact of unemployment and we as a field don’t have a good way of dealing with that”*

—interview respondent from a workforce development organization.

Exposure to trauma was mentioned as being a significant mental health issue associated with those who experience underemployment and unemployment. Trauma was described as a consequence that they believe is associated with living in long-term poverty. Organizations in workforce development are seeing an increasing need to be “trauma-informed” in their work with issues related to underemployment and unemployment and are now providing trauma-informed training for their direct service providers, especially when dealing with young adult workers. Respondents in workforce development and in industry concurred that the lack of knowledge of trauma history/experiences for marginalized populations impacts strategies to bring them (these populations) into the labor workforce.

*“People don’t sleep because they work back to back jobs. People use all sorts of stimulants, everything from drinking lots of soda and pop to other substances to keep going when they have to work multiple jobs………..I think you have issues of people’s security when you have to really think about how you manage your childcare when you’re working third shift and there’s no third shift childcare in this country. But just the running at 200% all the time, sort of no sleep, the precariousness of that has effects on people’s health”*

—interview respondent from a worker advocacy group.

*“I think one of the things that we do poorly as a system is address the health needs of—again, we meet people where they’re at. Often when we meet them, they are having health problems often related to the thing we’re trying to help them with. And we don’t have a good way of dealing with that”*

—interview respondent from a workforce development organization.

### 3.4. Other Challenges Expressed by Stakeholders

#### 3.4.1. Lack of a Common Agenda and Aligned Goals of Multiple Stakeholders Creates a Burden for All in Accomplishing Goals

Several interview respondents mentioned that it is challenging to align goals with local and political agendas and navigate a cluttered system.

*“…one of the things I’ve struggled with…is there’s so many different nonprofit groups out there……depending on who you talk to within government, everybody has their own pet project or their own organization that they have an allegiance to or a connection to”*

—interview respondent from industry.

*“everybody has to have their own program at the federal level, and there are 2000 competing programs out there. And we’ve made it so hard to navigate the system”*

—interview respondent from a skills coalition group.

Participants bemoaned the lack of sustained political will in advocating for workers and described how several US policies and practices favor powerful employers.

*“As far as things boosting labor standards and enforcement and unionization… those things have really honestly faced no support from (Congress) … It’s been sort of a both-sides-of-the-aisle are problematic.”*

—interview respondent from a policy group

#### 3.4.2. Current Workforce Development and Education Systems Lack the Coordination and Scale Needed to Support the 21st Century US Workforce

Respondents also believed that people are getting further and further left behind because community colleges (or higher education more broadly), vocational education, work-based apprenticeships, public schools, and second chance workforce systems have so many fewer resources than before.

*“The US invests at lower rates than every other industrialized country in workforce programming limitations…[…]… funding for the Workforce Innovation and Opportunity Act (WIOA), our public workforce system, has declined 40% since 2001”*

—interview respondent from a skills coalition group.

*“…[…]… there are far more people that have a college degree than we have jobs that require a college degree”*

—interview respondent from a policy group.

*“Not just giving businesses tax breaks but what does that mean about developing the infrastructure that this industry needs to survive…. the workforce being one of them? …..[…]… we don’t have industrial policy, we don’t have any kind of workforce strategy at all.[…]….we have built these best practices into policy like demand driven, sector driven, employer driven. But that’s still very individualized and neoliberal”*

—interview respondent from a workforce development organization.

Participants also described the increasing need for support of industry–sector partnerships at the local level and the need for leadership and collaboration to bring stakeholders together.

*“The role of industry and sector partnerships at a local level, these local communities of stakeholders, of local businesses, of community and technical colleges, and community-based organizations, of TANF agencies of the different folks that are interacting with and serving workers and employers is really how workforce development can be done really well. There’s good data on the fact that sectoral approaches have been successful in leading to employment and earnings outcomes. But these are the kind of partnerships that are difficult to support and difficult to sustain without federal or state investment in doing so”*

—interview respondent from a skills coalition group.

#### 3.4.3. Need for Strong Public Policies and Programs to Ensure Stability, upward Mobility, and Health Equity

Active labor market policies (ALMP) such as skills training programs or job creation measures may offer a ‘‘functional equivalent’’ to employment, providing, for example, ‘‘social contacts, … a clear time structure and … the feeling of participating in a useful collective purpose’’ [113]. Voßemer et al. argues that such policies should also “equip the unemployed with a feeling of control counteracting the restrictions on individuals’ agency” [114]. A second benefit of such programs is that they may function to provide “some of the social rewards of employment”, and “counteract the loss of human capital and improve unemployed individuals’ chances for quick and adequate re-employment” [114]. Strandh [115], Gundert and Hohendanner [116], and Wulfgramm [114] all discuss how the positive effects of training or job creation programs depend on their resemblance to regular employment, their response to workers’ needs, and participants’ perceptions. Importantly, not all ALMP are alike, and “measures that have an enforcing character and are perceived as paternalistic may not improve unemployed persons’ health” [113,114]. Despite these limitations, Voßemer et al. concede two additional hypotheses about the moderating effects of labor market policies: the more support through active labor market policies, the weaker the negative effect of unemployment on well-being and health; and the more support through active labor market policies, the weaker the negative effect of insecure jobs on well-being and health [114].

Poor health related to underemployment and unemployment has historically been ignored in economic policy discussions at the federal and state levels in the United States [117]. Voßemer et al. argues that the link between unemployment and health is “weaker in countries that provide generous unemployment benefits in terms of coverage or eligibility, duration, and wage replacement” [114]. He finds that “in these countries, most unemployed individuals receive benefits that compensate for the income loss for a comparatively long duration” and “therefore, the unemployed are able to afford a search for adequate reemployment, meaning that they do not have to fear any lasting negative career consequences” [114]. Two hypotheses anchor this research: the more generous the unemployment benefits, the weaker the negative effect of unemployment on well-being and health; and the more generous the unemployment benefits, the weaker the negative effect of insecure jobs on well-being and health [114].

Six out of nine respondents discussed the need to develop clearer messages about the experiences of marginalized individuals and communities and advocate for them, stating that there is increasing invisibility of those who are disconnected from the labor market, and that people are “on their own” in solving the problem of being disconnected from the labor market. Other respondents implicated system and public policy failures, stating that policies do not address problems with solutions that incentivize action to increase access for those disenfranchised from the labor market.

*“(When it comes to childcare) how people make it work is beyond me. I don’t understand as a society how we have not figured out how to not make this a burden on individuals because it crosses race lines, it crosses class lines. So, definitely childcare, in particular for women who are on the low spectrum of the income bracket, it’s make it or break it…if you are a logical, rational person and you are making minimum wage, you should not work because childcare costs more than what—let’s just do the numbers, right?”*

—interview respondent from a workforce development organization.

Overall, respondents described how the lack of a stable financial platform impacts people’s ability to access healthcare, education, food, and childcare, and supported the idea of some universal benefits.

*“There is also a gap in planning and understanding and thinking about what the social needs are and then carrying schooling and training to fulfill those needs. And that has to be a partnership, not just between private sector but also public sector because all that has to be paid for”*

—interview respondent from a skills coalition group.

## 4. Discussion

The following discussion is framed around how the findings aligned or challenged our initial understanding of the issues and the implications for the public health sector.

### 4.1. Underemployment and Unemployment Are Symptoms of Larger Problems

While on the surface underemployment and unemployment appear to be the problem, evidence from the literature and the interviews highlight the “wicked” [34,118,119,120] nature of these problems, with multiple macro-level drivers underlying the surface symptoms, including the changing nature of work, a dynamic labor market, inadequate enforcement of labor protection standards, declining unions, wage depression, and weak political will interacting with multiple social determinants of health. Additionally, the US work ecosystem is characterized by multiple stakeholders in government institutions, labor, economics, policy, workforce development, advocacy, industry, and education, who are involved in complex and unpredictable interactions. Therefore, similar to other public health problems, such as housing, access to healthcare, and obesity, it poses an adaptive challenge where there is no one, known solution to the problem, or where there are too many solutions but no clear choices. Preliminary evidence of the health, economic, and social impact of underemployment and unemployment, along with the relevance of these issues in global discussions regarding the future of work and health equity, make these “public health concerns”. Addressing these “wicked” problems in a sustainable way will require a systems perspective, taking into account the complex interrelationships and divergent perspectives of the key stakeholders to design effective interventions [34].

Therefore, there is a need to increase awareness across government, business, education, and community groups that underemployment and unemployment are not only economic problems, but also public health problems, and that a concerted national, systemic effort is needed to address both. Without intervention, a growing share of workers in the United States will experience unemployment, precarious employment and/or continue to feel underemployed, regardless of occupation, which may result in an increase of adverse social, emotional, mental, and physical health effects among workers and within the workforce. However, it is important that initiatives aimed at addressing the root causes of underemployment and unemployment occur at multiple social ecological levels. Often times, interventions focus on “downstream” behaviors, such as the recent focus on enhancing or expanding skills training programs, without acknowledging and addressing other inequities in education, funding, access, and job quality/pay. These initiatives do not acknowledge or address the “upstream” inequities, and therefore will not serve as a good strategy for the future. Regardless of whatever solution is proposed, it is important to recognize that the US economy is incredibly complex and varied: “with 1100 classified industries spread across more than 300 metro areas, the US economy is too diverse and unwieldy to fully assess skills gaps for the entire country”, while “different regions [are] facing different workforce challenges” [121]. This diversity requires the “development of regional strategies grounded in local data and local context about education providers, workers, and the needs of businesses” [121].

Finding solutions to complex issues like underemployment and unemployment will require coordination among multiple stakeholders, including policymakers, government agencies, educators, and employers, all of whom must be committed to implementing a common, shared agenda. Systems thinking is, by most definitions, more than just collective action; the ‘thinking’ part means integrating diverse perspectives to arrive at a deeper understanding of the complex dynamics and vicious cycles generating and regenerating wicked problems, with people able to propose interventions that generate virtuous cycles operating in a more benign direction [122]. This involves examining how macroeconomic shifts affect local and family economies, with consequences for the material basis of well-being, and also how shared (but not universal), persistent cultural models connecting employment, material success, status, life satisfaction, and self-esteem have implications for mental as well as physical health. Organizations typically do not use a systems thinking approach [35] in developing and designing programs and interventions. Fostering partnerships and networks among these stakeholders, and facilitating the adoption of systems practice-capability in order to think and act systematically [122], may lead to the identification of gaps and potential solutions in practice and help formulate policies related to underemployment and unemployment.

### 4.2. Gaps in Data and Research Will Need to Be Addressed in Order to Realize the Full Magnitude and Impact of Underemployment and Unemployment

There is a need for high quality research and reliable data to help improve the measures of underemployment and unemployment, not only at the national level, but at the local level, by community, race, gender, and disability status. This means recognizing other indicators and systems for tracking and reporting the “real” unemployment rates, developing standard definitions for underemployment and collecting, analyzing, and reporting underemployment data for key demographic groups, and, most importantly, finding ways to disseminate this information to sensitize the public, employers, policy makers, and workforce development organizations. It also means that public health data collection systems need to do a better job of incorporating work-related variables [123,124]. This will require a blurring of the construct of work-related/nonwork-related exposures and outcomes that has served as an artificial line of demarcation between occupational health and the rest of public health research for years [125,126]. For example, the influence that a job, or lack thereof, has on health goes beyond physical, emotional, and social conditions at work. Indeed, one’s job or career exerts a significant influence over other aspects of life that contribute or detract from an individual’s health and that of their family, such as income, social status, housing, access to healthcare, and free time to spend with family and friends [127]. As a result, work is seen as a principal mechanism for securing the needs to address health inequities and provide a concrete social location for influencing other social determinants of health as well [123,128,129]. 

There is also a need for standardization of other terms and measures used in the current labor economy. Over the past decade there has been an increasing conversation about different employer—employee relationships, such as contingent work, precarious work, and other nonstandard work arrangements, such as temp work and gig work. Maddocks argues there exists greater need to distinguish the concepts of underemployment from other concepts such as nonstandard work, precarious employment, and job dissatisfaction [43]. Whereas nonstandard work has typically been defined “in contrast to a standard employment relationship where employees work full-time year-round, receiving statutory benefits, and have the expectation that they will be employed indefinitely”,… “the concept of precarious employment has been put forth by sociologists to describe some forms of non-standard work, including part-time work or variable work schedules, reduced job security, lack of union protection, low wages, lack of employer sponsored benefits, or stressful psychosocial relationships and working conditions” [43,130]. Accordingly, these parameters are designed to identify jobs that espouse precarious work relationships where the worker has “little or no legal or regulatory protection against experiences such as the potential loss of their job, a decline in their hours of work, or control over the labour process” [43,131,132]. Clearly, some of the attributes of precarious work are very similar to those used to identify underemployment, such as part-time work or low wages. However, while precarious jobs are expected to have negative consequences for all workers who hold them, the underpinnings of underemployment are more complex. Maddocks claims that underemployment is “a condition for the individual and not necessarily an inherent feature of the job itself” and that “underemployment indicators identify jobs that are unsatisfactory for the individual in comparison to a previous job or expectations”. This means, for example, having part-time employment when full-time work is desired, or earning lower wages than in a previous job rather than simply having part-time work at low wages. Some individuals may desire part-time, flexible work contracts, or may be employed in a precarious employment relationship without being underemployed [43]. Alternate measures of the labor market reported by respondents in the interviews were similar to those found in the literature. Respondents in all interviews stated that they do not believe we have good measures or understanding of both underemployment and unemployment, noting that measuring underemployment is particularly complicated. The labor force participation rate, along with the unemployment rate, could provide a more accurate picture of the job market.

The literature examining the links between unemployment and negative health outcomes spans almost forty years and explores contexts across continents. Substantial statistical evidence has been gathered showing the link between the lack of work in its various forms and workers’ physical and mental health problems. Overall, the literature indicates gaps in research on unemployment and specific types of underemployment, and on indicators of health and well-being in specific population groups, such as young workers, women, and low-income people of color living in neighborhoods who are disproportionately burdened by job loss. Key informant interview respondents identified two key barriers in considering health outcomes as they relate to their work in addressing underemployment and unemployment: (1) firstly, while most respondents were aware of the mental and physical impacts of underemployment and unemployment, they did not have any access to, or use for, health and well-being related tools in their program planning/thinking in workforce development, policy development, skills training, and industry; (2) secondly, respondents acknowledged the need for being “trauma-informed” in addressing prior trauma and mental health issues in workforce development programing and delivery, but were challenged by the lack of available strategies and tools. Organizations have started to develop in-house trainings and tools to address this gap; however, these programs are already underfunded, and investing in the development of these additional tools and training programs to address these barriers increases the strain on their systems and employees.

Other data needs that may promote an improved understanding of the problem include data on the impact of other nonstandard employment conditions on health and well-being, data on the role of education and training in skills gap and/or mismatch, research on sustainable wages and employment, and research on the role of the absence or presence of unions and the enforcement of labor standards.

### 4.3. Need for Strategic Funding to Foster Scaling-Up and Translation of Successful Models of Initiatives and Partnerships between Employers–Educators, Employers–Workforce Development, Labor Management–Advocacy in Workforce Development, Education, Research and Policy

Place-based strategies can play a critical role in improving the health and well-being of workers and communities. Often such efforts do not get reported in the literature. For example, interview respondents described initiatives where a local skills training program partnered with other local health agencies to provide childcare stipends for women in a construction apprenticeship program, where a workforce development program provided temporary housing for people who are homeless and unemployed, and where a community college system representative described how they negotiated a stipend aligned to federal standards for students enrolled in an “earn and learn” program. These stories need to be told, as they provide rich information on models and enterprises at the community level that can contribute to a comprehensive list of best practices for different economic contexts.

While there is a continued emphasis on traditional research and increasing support for translational research [133], there is also a need to support those kind of practice-based research partnerships because they are really important for providing stability, and because workforce and jobs are inherently local. Evidence produced by academic research institutions is rigorous and often shaped by the traditional values of the academy [134]. However, such research may miss opportunities to understand local contexts and produce actionable findings. Research–practice partnerships could provide policy insights and data that can facilitate rigorous and groundbreaking research; likewise, it could also provide practitioners with easy access to and timely use of research evidence [134]. Opening the door for funding practice-based research partnerships may promote collaboration between researchers and multisector stakeholders in the work ecosystem to define a more holistic agenda that aligns future research, practice, and policy. For example, a systematic analysis and evaluation of current national and local policies, funding, partnerships, and implementation strategies to boost the economy and employment may give some insights into what policies are worth scaling up. What data are being used in these decision-making processes? Workforce development organizations have developed innovative partnerships with employers to create pipelines for jobs. Right now, it is unclear how these best practices and innovative models are being evaluated for their success, how job quality (or good work, or good jobs) is being defined, how such information is being gathered and monitored, and how data (if any) are being used to strengthen the existing funding and policy structures. If we want to be strategic in scaling up these efforts, there is an urgent need for systematically researching, documenting, supporting, and disseminating these efforts.

## 5. Conclusions and Recommendations

The purpose of the environmental scan was not to be an exhaustive exploration of underemployment and unemployment and their outcomes (health and other). Rather, it was intended to be an exploration of evidence relevant to the field of public health and its partners that ought to be included in discussions of future strategic agendas and initiatives across public health, government agencies, industries, community colleges, universities, trade and business associations, labor organizations, and other stakeholder groups. Although the literature review was not a systematic review, it was a comprehensive review of close to 100 articles. This study could have benefitted from additional interviews that captured perspectives of a diverse spectrum of representatives from state, local, federal agencies, trade and business associations on underemployment and unemployment, and workers; however, the information gathered during the interviews provides very rich information, with convergent themes emerging across a diverse array of stakeholders, that need to be explored further. Finally, this study did not include any direct research on the health impacts of COVID-19.

The impacts of unemployment, underemployment, gig work, precarious jobs, and the changing nature of work is gaining traction around the world amidst the climate of economies struggling with the inconsistencies of the labor market in the post-pandemic world, the impending challenges for employers and workers with the rise of artificial intelligence (AI) and robotics, and the uncertainty surrounding the future of work. While the empirical literature has established that job loss or unemployment results in significant deterioration in well-being, researchers argue that satisfaction with employment is a key ingredient differentiating employment and unemployment experiences. In the wake of the global pandemic and economic crisis, there is a unique window of opportunity to raise consciousness and highlight the urgency of delivering quality jobs along with social protection and respect for rights at work to achieve sustainable, inclusive economic growth and eliminate poverty. Recent months have seen revived discussions, by lawmakers and administration officials, of initiatives to raise the minimum wage, strengthen unions and worker protection standards, and provide a universal basic income and full employment [60]. Laudable as these efforts may be, it is highly likely that the United States could still fall short of actually bringing about the adoption and implementation of these policies and practices because, as a society, we still do not have a shared understanding of the significance of workforce health and well-being to our nation’s health.

Workforce health is population health, so what is the value of the health and well-being of the US worker and the workforce? Further, what is the value of decent work in public health policy, or public policy? Public health professionals at the intersection of occupational and population health are in a unique position to address worker and workforce health and well-being beyond the workplace by leading strategic collaborations that integrate health and economic development (work) across government agencies, and in partnership with industries, community colleges, financial institutions, universities, trade and business associations, labor organizations, and other stakeholder groups to support and promote a human-centered, equitable, and decent future of work—a future of work where every worker is healthy and able to achieve their full potential by engaging in meaningful work.

**Figure 2:**
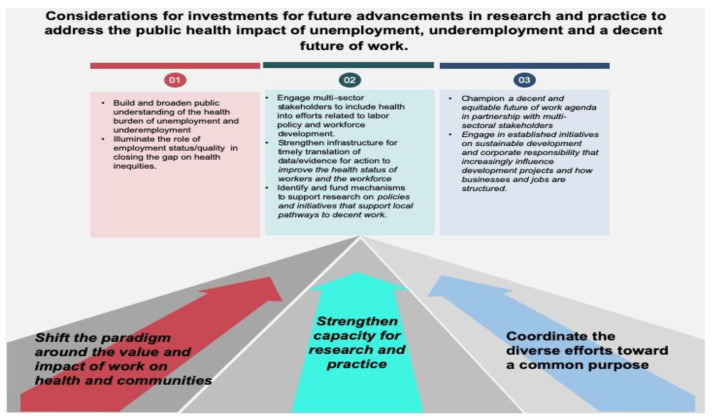
Considerations for a common framework to address the public health impact of underemployment and unemployment, and promote a decent future of work.

Considerations for future investments in research and practice, and a common framework to address the public health impact of underemployment and unemployment, and promote a decent future of work, are described in Figure 2.

- *Shift the paradigm around the value of work and its impact on individual health and communities, by investing in broadening public understanding of the health burden of underemployment and unemployment.* As researchers and practitioners in the field of occupational health, we understand that although social determinants of health (SDoH) “are the conditions in which people are born, grow, live, work and age”, what people do for work and at work does not figure in larger discussions related to SDoH [128]. Despite the conceptual acknowledgment that work influences health through numerous pathways, it remains largely absent from examinations of health inequities in the United States [123]. Public health needs to illuminate the role of employment quality and status in closing the gap on health inequities.
- *Strengthen capacity for research and practice by engaging the multisector stakeholders that public health may not routinely engage with, such as workforce development, labor policy, and corporate sustainability.* Inclusion of health into efforts related to labor policy development and other economic development agendas can add value to discussions regarding the burden of the problem on health and the healthcare system, economic investments in skills training and social benefits, and improve the health status of workers and the workforce [124]. Strengthening infrastructure for timely use of data/evidence for action in labor policy and workforce development could ensure the jobs they create are designed to contribute to healthier workers and communities. New funding mechanisms are also needed to support practice-based research partnerships that address institutional and systems-level facilitators and barriers to the innovation and implementation of policies and initiatives in order to support local pathways to decent work.
- *Coordinate the diverse efforts in the work ecosystem towards a common purpose and a shared agenda for a decent future of work.* Currently, diverse agendas and potential misalignment of multiple stakeholders, the overall complexity of the problem, a lack of metrics and actionable data, and a lack of leadership and a common language are limiting the ability of stakeholders in the work ecosystem to collectively address the impact of underemployment and unemployment, and assess the future of work more generally [8]. Under the public health model, increasing the number and quality of jobs can serve as a prevention strategy by increasing economic security, self-esteem, and social connectedness. “*Public Health 3.0 A Call to Action for Public Health to Meet the Challenges of the 21st Century*” [135] refers to a new era of enhanced and broadened public health practice that goes beyond traditional public health department/agencies functions and programs. Cross sectoral collaboration is inherent in Public Health 3.0. As such, labor policy that is a part of health policy, and a discussion of job quality or decent work, not just job quantity, could help public health professionals engage in more established initiatives on sustainable development and corporate responsibility that increasingly influence development projects and how businesses and jobs are structured [124]. Public health needs to champion an agenda in partnership with multisector stakeholders to integrate workforce health and well-being into labor and economic development agendas across government agencies and industry.
